# High-dose sequential epirubicin and cyclophosphamide with peripheral blood stem cell support for advanced breast cancer: results of a phase II study

**DOI:** 10.1054/bjoc.2001.2069

**Published:** 2001-09-01

**Authors:** P H Cottu, J M Extra, M Espie, J P Marolleau, A de Roquancourt, J Makke, J M Miclea, V Laurence, D Mayeur, F Lerebours, C Cuvier, M Marty

**Affiliations:** Department of Medical Oncology, Hôpital Saint-Louis, 1 av Claude Vellefaux, Paris, 75010, France; Department of Cell Therapy, Hôpital Saint-Louis, 1 av Claude Vellefaux, Paris, 75010, France; Department of Pathology, Hôpital Saint-Louis, 1 av Claude Vellefaux, Paris, 75010, France; Department of Medical Oncology, Hôpital André Mignot, Le Chesnay, 78000, France

**Keywords:** breast cancer, advanced, chemotherapy, high-dose

## Abstract

The aim of this study was to evaluate the feasibility of a high-dose intensity and high-dose density multicycle epirubicin and cyclophosphamide regimen with peripheral blood stem cells (PBSC) and haematopoietic growth factor (G-CSF) support in advanced breast cancer patients. From August 1994 to September 1999, 56 breast cancer patients (8 stage IIIB and 48 stage IV) received 205 courses of cyclophosphamide 3 g m^−2^and epirubicin 100 mg m^−2^every 14 days. G-CSF 5 μg kg^−1^day^−1^was administered from day 3 to neutrophil recovery. 4 courses were planned. PBSC were collected after course 1, and reinfused after courses 3 and 4, with ≥ 2 × 10^6^CD34+ PBSC kg^−1^required for each reinfusion. 48 patients (86%) received all 4 planned courses. Early withdrawal was consecutive to infectious complications (*n*= 4), severe asthenia (*n*= 3), haemorrhagic cystitis (*n*= 1). A median number of 10.8 × 10^6^CD34+ PBSC kg^−1^(range, 3–80) was harvested with 1 or 2 apheresis in 48 patients (94%). Median relative dose intensity was 91.3% (range, 72–102%). Grade 4 neutrophil toxicity was observed in 100% of patients. Febrile neutropenia was observed in 40% of courses (median duration 2 days). Red blood cells and platelets had to be transfused in 54% and 27% of courses, respectively. There were no toxic deaths. Objective response rate was 69% in stage IV patients (31/45 evaluable pts), with a 16% complete response rate. Their median progression-free and overall survivals were 22.5 and 37 months, respectively. This epirubicine-containing high-dose regimen appeared feasible, albeit with high toxicity. Time-related progression parameters exceed commonly reported ones. Controlled studies of upfront sequential high-dose chemotherapy are still needed to evaluate its real benefit. © 2001 Cancer Research Campaign


*British Journal of Cancer* (2001) **85**(9), 1240–1246
© 2001 Cancer Research Campaign
doi: 10.1054/ bjoc.2001.2069, available online at http://www.idealibrary.com on IDEAL

http://www.bjcancer.com

### High-dose sequential epirubicin and cyclophosphamide with peripheral blood stem cell support for advanced breast cancer: results of a phase II study

**PH Cottu[1], JM Extra[1], M Espie[1], JP Marolleau[2], A de Roquancourt[3], J Makke[2], JM Miclea[3], V Laurence[1], D Mayeur[4], F Lerebours[1], C Cuvier[1] and M Marty[1]**

[1]Department of Medical Oncology, Hôpital Saint-Louis, 1 av Claude Vellefaux, 75010 Paris, France; [2]Department of Cell Therapy, Hôpital Saint-Louis, 1 av Claude Vellefaux, 75010 Paris, France; [3]Department of Pathology, Hôpital Saint-Louis, 1 av Claude Vellefaux, 75010 Paris, France; [4]Department of Medical Oncology, Hôpital André Mignot, 78000 Le Chesnay, France

**Summary** The aim of this study was to evaluate the feasibility of a high-dose intensity and high-dose density multicycle epirubicin and cyclophosphamide regimen with peripheral blood stem cells (PBSC) and haematopoietic growth factor (G-CSF) support in advanced breast cancer patients. From August 1994 to September 1999, 56 breast cancer patients (8 stage IIIB and 48 stage IV) received 205 courses of cyclophosphamide 3 g $m^{-2}$ and epirubicin 100 mg $m^{-2}$ every 14 days. G-CSF 5 µg $kg^{-1}$ $day^{-1}$ was administered from day 3 to neutrophil recovery. 4 courses were planned. PBSC were collected after course 1, and reinfused after courses 3 and 4, with $\geq 2 \times 10^6$ CD34+ PBSC $kg^{-1}$ required for each reinfusion. 48 patients (86%) received all 4 planned courses. Early withdrawal was consecutive to infectious complications (*n* = 4), severe asthenia (*n* = 3), haemorrhagic cystitis (*n* = 1). A median number of $10.8 \times 10^6$ CD34+ PBSC $kg^{-1}$ (range, 3–80) was harvested with 1 or 2 apheresis in 48 patients (94%). Median relative dose intensity was 91.3% (range, 72–102%). Grade 4 neutrophil toxicity was observed in 100% of patients. Febrile neutropenia was observed in 40% of courses (median duration 2 days). Red blood cells and platelets had to be transfused in 54% and 27% of courses, respectively. There were no toxic deaths. Objective response rate was 69% in stage IV patients (31/45 evaluable pts), with a 16% complete response rate. Their median progression-free and overall survivals were 22.5 and 37 months, respectively. This epirubicine-containing high-dose regimen appeared feasible, albeit with high toxicity. Time-related progression parameters exceed commonly reported ones. Controlled studies of upfront sequential high-dose chemotherapy are still needed to evaluate its real benefit. © 2001 Cancer Research Campaign

**Keywords**: breast cancer; advanced; chemotherapy; high-dose

Curability of advanced breast cancer, either locally advanced or metastatic, remains poor. With hormonal treatment and systemic polychemotherapy, relapse-free survival (RFS) is 35% and overall survival (OS) is 40% at 5 years for stage IIIB patients. For stage IV patients, OS remain below 20% at 5 years (Flamm Honig, 1996). Several attempts have been made to further improve relapse-free, progression-free (PFS) and overall survival in both locally advanced and metastatic breast cancer, based on preclinical and biological studies. A linear dose–response relationship has been demonstrated particularly for alkylating agents which remain among the most active drugs for breast cancer (Skipper et al, 1964). Many authors stressed the importance of cell repopulation in the resistance of cancer to chemotherapy. Differences in the cell death and repopulation curves (i.e. non-exponential versus exponential) have led to the concept of timing and schedule as important parameters for the efficacy of cytotoxic agents. The shortening of the interval between the courses of chemotherapy might therefore be at least as beneficial as the dose increase of the same agents (reviewed in Gilewski and Norton, 1996). The increased dose intensity or dose density of combination chemotherapy regimens may be achieved by either increasing the doses of the drugs, or shortening the intervals between courses, or both.

However, increasing the doses of cytotoxic agents was limited by high haematological and infectious morbidity and mortality. After 1990, haematologic support with haematopoietic growth factors (G-CSF and GM-CSF) and blood cells progenitors derived from the bone marrow (ABMT) or from peripheral blood (PBSC) became widely available to circumvent the dose-limiting haematological toxicity and infectious complications. Many studies of high-dose chemotherapy (HDC) with G-CSF and ABMT/PBSC have been published, including studies dealing with HDC multicycle regimens (Rodenhuis et al, 1996; Vahdat and Antman, 1997), and 3 randomized studies (Peters et al, 1996; Lotz et al, 1999; Stadtmauer et al, 2000). Most of these regimens included only alkylating agents (Peters et al, 1996; Rodenhuis et al, 1996; Antman et al, 1997; Vahdat and Antman, 1997; Stadtmauer et al, 2000), only 3% of them including mitoxantrone in the conditioning combination (Antman et al, 1997; Vahdat and Antman, 1997; Lotz et al, 1999). A fourth randomized study had been published earlier (Bezwoda et al, 1995), but an international audit has demonstrated that 'the multiple publications of this study (...) do not report verifiable data' (Weiss et al, 2001). The data from this study will not be furthermore commented in the paper.

*Received 20 November 2000*
*Revised 10 July 2001*
*Accepted 20 July 2001*

*Correspondence to:* PH Cottu

Presented in part at the 33rd Annual Meeting of the American Society of Clinical Oncology, Denver, CO, 1997.

Although anthracyclines are the reference agents in advanced breast cancer and a clinical dose–response relationship has also been demonstrated in advanced breast cancer with epirubicin (Bastholt et al, 1996), no regimen had doxorubicin or epirubicin. In 1986 we began an open phase II trial of an accelerated protocol for stage IIIB–IV patients, associating epirubicin 75 mg $m^{-2}$ and cyclophosphamide 1200 mg $m^{-2}$ every 2 weeks, given without any haematopoietic support for 6 consecutive courses. The patients were to be retreated at day 14 whatever the leukocyte count in the absence of febrile neutropenia. This induction protocol was to be followed by 6 months of conventional maintenance combination chemotherapy. Results have already been reported, confirming its feasibility, and underscoring its efficacy in terms of PFS and OS (Culine et al, 1993; Extra et al, 1995; Cottu et al, 1999). Other investigators have proposed a similar approach including haematopoietic growth factor support, which did not significantly improve the delivered dose intensity nor the clinical results (Piccart et al, 1995; Lalisang et al, 1997).

Since most very high-dose studies have been limited to one or two treatment courses which usually did not include anthracyclines, and based on our previous results of a dose dense epirubicin–cyclophosphamide combination, we devel–oped a further incrementalistic anthracycline-containing chemotherapy regimen, consisting of 4 courses of high-dose 2-weekly chemotherapy regimen including epirubicin and cyclophosphamide, supported by haematopoietic growth factors and PBSC, as induction therapy for stage IIIB–IV breast cancer patients, followed by conventional maintenance chemotherapy. The main objectives were to assess the feasibility of this approach, to determine acute haematological and extra-haematological toxicity, and long-term side-effects, and to evaluate the potential efficacy, focusing on the PFS and OS results. We report here the results of our experience in 56 patients.

### PATIENTS AND METHODS

### Patients

Women with histologically proven breast adenocarcinoma were included. Stage IV and IIIB (with T4d tumours) patients, with evaluable and/or measurable disease, were to be included. Other inclusion criteria were: age 18–55, PS 0–1, adequate left ventricular ejection fraction (LVEF) as assessed by gated radionuclide scan (i.e. ≥ 50%), normal liver function (i.e. transaminases ≤ 1.5 N, alkaline phosphatases ≤ 1.5 N, total bilirubin ≤ N), normal renal function (serum creatinine clearance > 80 ml $min^{-1}$), normal blood count before the first course. The possibility of close follow-up was a compulsory accrual clause. Metastatic patients who had received adjuvant chemotherapy were not excluded if the cumulated doses of epirubicin and/or doxorubicin were under 400 mg $m^{-2}$ or 300 mg $m^{-2}$, respectively. Extensive disease evaluation was performed, including complete physical examination, mammograms, isotopic bone scan, abdominal ultrasound exploration, bone marrow biopsy, serum tumour markers. Chest and abdominal computerized tomography (CT scan) were done when indicated. Exclusion criteria were: age over 55, PS ≥ 2, metastatic bone marrow, inadequate liver, renal or cardiac function (LVEF < 50%), elevation of serum tumour marker(s) as sole evidence of disease, previous chemotherapy for metastatic or advanced disease, or history of any other malignancy. All patients had given informed consent.

### Chemotherapy

Chemotherapy consisted of epirubicin 100 mg $m^{-2}$ day 1 in a short i.v. infusion, and cyclophosphamide 1500 mg $m^{-2}$ day 1 and day 2 (i.e. 3000 mg $m^{-2}$ per course) with equidose uromitexan in 1 hour i.v. infusion. Patients were hospitalised in the afternoon of day 1 and released in the morning of day 3. They received 4 litres of isotonic saline solution/24 h as continuous intravenous infusion for 2 days, along with corticosteroids and serotonin antagonists as antiemetic medication. Furosemide 20 mg i.v. was administered if the 6 hours diuresis was under 800 ml. The treatment was to be resumed every 14 days, whatever the absolute neutrophil count. If the patient presented with febrile neutropenia, the treatment was resumed after 24 h of apyrexia. The treatment was delayed until recovery in case of persistent thrombocytopenia inferior to 30 000 $mm^{-3}$, transaminases elevation above 2.5 × N, or serum creatinine clearance under 80 ml $min^{-1}$. The patients were withdrawn from the study if any of the following complications occurred: persistent (> 7 days) febrile neutropenia, grade 4 infection, platelets < $30.10^9$ $1^{-1}$ at day 21, grade 3–4 non-haematological complication persistent at day 21. Platelets and red blood cells were transfused in an ambulatory setting if under $20.10^9$ $1^{-1}$, and haemoglobin under 8 g $dl^{-1}$, respectively, or as clinically indicated. 4 courses were planned before re-evaluation. Evaluation of response was assessed according to WHO criteria. For patients who had stage IIIB or stage IV at presentation breast cancer, local therapy including surgery and radiation therapy was performed as indicated. Pathological response was assessed separately for the breast and the axillary lymph nodes, and was divided into complete (no viable tumour cells), partial (post chemotherapeutic changes with persistent viable tumour cells) and no response, and node-positive or node-negative, respectively.

The dose intensity (DI) was computed for every patient who had completed the 4 courses. No dose reduction was allowed, and the treatment was reconducted on the 14th day whatever the neutrophil count, except if the patient presented with any of the complications previously described. Thus, only the number of days between day 1 of course 1 (Date C1) and day 1 of course 4 (Date C4) was used in the calculation with the following formula: DI = [Date C4 – Date C1] / 42, where 42 represents the minimal duration between day 1 of course 1 and day 1 of course 4 (Shapiro et al, 1997).

Maintenance chemotherapy consisted of a 6 months outpatient alternative regimen including CMF (months 1–4), a combination of 5-fluorouracil, methotrexate and vincristine (months 2–5), and 5-fluorouracil/adriamycin/cyclophosphamide (FAC) (months 3–6). Tamoxifen was added to patients whose primary tumour expressed estrogen receptors, provided they had not received and/or failed adjuvant hormonal therapy. Patients with progressive disease during or after the completion of the study regimen were given vinorelbine- or, more recently, docetaxel-based regimens. Patients with CNS disease were not given radiation therapy, unless they evidenced progressive and/or symptomatic disease.

### Cellular therapy

Recombinant human granulocyte-colony stimulating factor (G-CSF) at a daily dose of 5 μg $kg^{-1}$ given subcutaneously was used from day 3 until neutrophil recovery (i.e. > 1000 $mm^{-3}$) or until day –2 of the following course, for the 4 courses. Peripheral blood stem cells (PBSC) were mobilized after the first course by the

© 2001 Cancer Research Campaign combination of chemotherapy and G-CSF. Patients had complete blood count measured every other day, and cell apheresis began during the rebound recovery period. Collections were performed in order to obtain a total of $4 \times 10^6$ CD34+ PBSC $kg^{-1}$, which was considered as necessary for an appropriate engraftment, i.e. $2 \times 10^6$ CD34+ PBSC $kg^{-1}$ for course 3 and 4. The apheresis was repeated until the scheduled $4 \times 10^6$ CD34+ PBSC $kg^{-1}$ were collected, and if necessary was repeated after course 2. The apheresis was delayed if the patient had febrile neutropenia. For each apheresis, a median of 10 l of blood were processed at a flow rate of 40–60 ml $min^{-1}$. Acid-citrate-dextrose (ACD-A) was used as the inlet AC ratio. The quantity of progenitor cells was assessed by numeration of CD34+ cells. CD34+ cells count assay was performed after staining with HPCA2 (a monoclonal antibody against CD34+ antigene). Fluorocytometry was performed on a fluorescent automatic cell sorter. 50 000 events were acquired. The absolute number of CD34+ cells was derived by multiplying the percentage of CD34+ cells and the absolute number of mononucleated cells. Apheresis collections were cryopreserved in a minimum of 4 bags. The cells were volume adjusted to a final concentration inferior to $1.5 \times 10^8$ WBC $ml^{-1}$. The final product containing 10% DMSO (dimethlyl sulfoxide) in autologous plasma was frozen according to standard methods and stored in liquid nitrogen. Before reinfusion, cells were thawed in the stem cell laboratory, and suspended in an albumin 4% and sodium chloride buffer. A cell count, a viability assay and a CD34+ assay were performed. Reinjection was done over 30–60 minutes in the clinical unit.

Epithelial cell contamination of the grafts was determined and will be described in a separate report (Dal Cortivo et al, 2001).

### Toxicity monitoring

The treatment was designed as an outpatient programme. All toxicities were graded according to the WHO criteria. Close clinical follow-up was done, including daily telephone contact with the patient when necessary. Complete blood count and biochemistry tests were monitored 2 or 3 times a week. Every patient received oral antibiotherapy with cefixime (200 mg twice daily) as soon as fever was higher than 38˚C. If fever persisted more than 24 h, or if overt signs of infections appeared, the patient was hospitalized for evaluation and intravenous broad-spectrum double antibiotherapy. Patients were also hospitalized if they presented persistent grade 3 extra-haematological toxicity (emesis, liver, renal and cardiac toxicity). Preventive treatment of mucositis consisted in multiple daily mouth washes with a solution of amphotericine B, chlorhexidine and 1.4% sodium bicarbonate. Cardiac toxicity was clinically evaluated after each course, and with a LVEF evaluation at the end of the 4 high-dose courses and of maintenance therapy.

### Objectives

The primary aim of the study was to assess the feasibility of a multi-cycle high-dose-intensity and dose-density regimen including epirubicin. The feasibility criteria we had pre-defined as acceptable were: appropriate leukapheresis for 2 engraftments, i.e. more than $4 \times 10^6$ CD34+ PBSC $kg^{-1}$; relative dose intensity greater than 80% in more than 80% of patients; treatment-related mortality inferior to 5%. Dose-limiting toxicities are described above. The secondary aim was the assessment of the efficacy of the treatment. The 4 courses were to be administered in at least 80% of the patients, and we expected less than 10% of patients with progressive disease at the end of high-dose treatment.

### Statistical methods

Descriptive statistics are reported as percentages and medians (Snedecor and Cochran, 1989). Overall survival and PFS were defined as the time from the day of the first course to death, disease relapse or disease progression, respectively. Progression of disease was defined as any progression of known metastatic site according to WHO criteria, or of emergence of a new site, including contralateral breast. Survival curves were constructed with the Kaplan and Meier product-limit method (Kaplan and Meier, 1958). Univariate analysis of prognostic factors for OS and PFS was conducted using the log-rank test (Snedecor and Cochran, 1989). Data acquisition for PFS and OS was done until January 31st, 2000.

### RESULTS

### Population

From August 1st, 1994 to September 1st, 1999, 56 patients were entered in the study. All patients but 2 were treated at Hospital Saint-Louis. Details of presentation are shown in Table 1. Median age was 43.4 (range, 23.5–57). The population included 8 patients with stage IIIB (T4d) carcinoma, 27 patients with stage IV at presentation, and 21 relapsing metastatic patients. This accounted for about 15% of all new stage IV patients who were referred to our centre in the same time span. 13 relapsing metastatic patients had received previous adjuvant chemotherapy, 8 of them with anthracyclines. Median disease-free interval for this subset of patients was 37 months (range, 13–98). A high proportion (67%) had 2 or more involved sites, and nearly the same proportion (65%) had at least one visceral metastatic site. Main metastatic sites for all stage IV patients were liver (40%), lymph nodes (40%), bone (38%), and lung (19%). 13 patients with stage IV disease at presentation and 6 stage IIIB patients underwent mastectomy and axillary dissection after the induction chemotherapy, and were evaluated for pathological response.

### Toxicity and feasibility

All patients, receiving a total of 205 treatment courses, were evaluable for feasibility and toxicity (Tables 2 and 3). Leukapheresis with G-CSF support was performed as planned (Table 2). The median day for collection was day 11 after course 1. One leukapheresis collection was sufficient in 40 patients (72%), and only 3 patients (5%) had 3 or more collections. The median number of collected CD34+ PBSC was $10.8 \times 10^6$ $kg^{-1}$ (range 3–80), and 54 patients (96%) had more than $4 \times 10^6$ CD34+ PBSC $kg^{-1}$. The relative dose-intensity was computed for the 48 patients who had completed the 4 courses. The resulting median relative dose-intensity was 91.3% (range 72–102). It is noteworthy that 80% and 94% of patients had a dose-intensity greater than 90% and 80%, respectively. Conventional maintenance chemotherapy, radiation therapy when indicated, and second-line therapy were feasible without any unusual toxicity (data not shown).

No treatment-related death has been recorded. The treatment had to be interrupted before completion of the 4 courses in 8 patients (14%): 1 patient with haemorrhagic cystitis after course 3,

 © 2001 Cancer Research Campaign

**Table 1** Population characteristics ($n$ = 56)

| | | Median | Range |
|---|---|---|---|
| Age at inclusion (years) | | 43.4 | 23.5–57 |
| Disease-free interval (months)[a] | | 37 | 13–98 |
| | | **n** | **%** |
| Stage at inclusion | IIIB (T4d) | 8 | 14 |
| | IV (presentation) | 27 | 48 |
| | IV (relapse) | 21 | 38 |
| WHO performance status | 0 | 39 | 70 |
| | 1 | 13 | 23 |
| | 2 | 4 | 7 |
| Menopausal status | pre | 47 | 84 |
| | post | 9 | 16 |
| Adjuvant chemotherapy[a] | With anthracyclines | 8 | 38 |
| | Without anthracyclines | 5 | 24 |
| | None | 8 | 38 |
| Metastatic sites[b] | Liver | 19 | 40 |
| | Lymph nodes | 19 | 40 |
| | Bone | 18 | 38 |
| | Lung | 9 | 19 |
| | Pleura | 3 | 6 |
| | CNS[c] | 3 | 6 |
| | Skin | 1 | 2 |
| | Other[d] | 3 | 6 |
| Number of site(s)[b] | 1 | 16 | 33 |
| | ≥2 | 32 | 67 |
| Number of viscera(e)[b] | 0 | 17 | 35 |
| | ≥1 | 31 | 65 |

[a]Refers to 21 patients who relapsed; [b]refers to 48 stage IV patients; [c]CNS: central nervous system. Includes brain, epidural and meningeal metastases; [d]1 ovary and 2 peritoneal metastases.

**Table 2** Feasibility results ($n$ = 56)

| | | *n* | | % |
|---|---|---|---|---|
| Number of courses | 4 | 48 | | 86 |
| | 3 | 4 | | 7 |
| | 2 | 1 | | 2 |
| | 1 | 3 | | 5 |
| Number of leukapheresis sessions | 1 | 40 | | 72 |
| | 2 | 13 | | 23 |
| | >2 | 3 | | 5 |
| Leukapheresis delay | | | | |
| Median (days) | | | 11 | |
| Range | | | 10–15 | |
| CD34+ | | | | |
| Median (× $10^6$/kg) | | | 10.8 | |
| Range | | | 3–80 | |
| ≥4 × $10^6$/kg | | 54 | | 96 |
| Dose intensity[a] | | | | |
| Median (%) | | | 91.3 | |
| Range | | | 72–102 | |
| ≥80% | | 45 | | 94 |

[a]Evaluated on the 48 patients who completed the 4 courses.

3 patients with persistent grade 3 asthenia after courses 2 (2 patients) and 3 (1 patient), and 4 patients with grade 3–4 infection after courses 1 (3 patients) and 3 (1 patient). One of the latter patients presented grade 3 oesophagitis and non-metastatic bowel invagination which required a 30 cm bowel surgical excision. Another patient had iterative febrile neutropenia episodes lasting longer than 7 days. The last 2 patients presented subcutaneous infectious cellulitis (*Staphylococcus aureus*), one of whom required surgical excision. Details of toxicity by course are given in Table 3. Briefly, grade 4 neutropenia was observed after 90% of courses and in 100% of patients. Median duration of grade 4 neutropenia was 4 days (range, 2–11 days). Febrile neutropenia requiring rehospitalisation was observed in 79 courses (39%), with a median duration of 2 days. Only 13 patients (23%) did not experience febrile neutropenia. Red blood cells had to be transfused in 104 courses (51%) and in 49 patients (88%). Platelets were transfused in 55 courses (27%), and in 32 patients (57%). Median duration of grade 4 thrombopenia was 3, 2, 4 and 3 days after courses 1, 2, 3 and 4, respectively. No significant change in the LVEF has been observed, neither after induction, nor at the end of maintenance therapy. Extra-haematological toxicity is shown in Table 3. No grade 3 liver or renal toxicity was recorded. An on-treatment decrease in the performance status was observed in 18 patients (32%), 3 of whom were withdrawn from the study. An improvement was observed in 5 patients (9%). 14 patients (25%) did not require rehospitalisation.

### Response and survival

45 stage IV patients who had received at least 2 courses were considered as evaluable for response. Clinical overall response rate was 69% (95% CI: 55.5–82.5). Complete response rate was 16% (95% CI: 5.3–26.7). Intent-to-treat analysis for the 48 metastatic patients showed an overall response rate of 64.5% (95%

© 2001 Cancer Research Campaign

**Table 3** Toxicity by course

| | | C1 (*n* = 56) | C2 (*n* = 53) | C3 (*n* = 52) | C4 (*n* = 48) |
|---|---|---|---|---|---|
| Neutrophils | | | | | |
| | Grade 4 (%) | 95 | 89 | 89 | 88 |
| | Median duration (days) | 4 | 4 | 6 | 5 |
| | Range | 2–8 | 1–9 | 2–11 | 2–10 |
| Fever | | | | | |
| | % | 30 | 28 | 30 | 37 |
| | Median duration (days) | 2 | 2 | 2 | 2 |
| | Range | 1–6 | 1–6 | 1–9 | 1–13 |
| Hospitalization | | | | | |
| | % | 29 | 35 | 40 | 53 |
| | Median duration (days) | 3 | 4 | 3 | 4 |
| | Range | 1–22 | 1–7 | 1–14 | 1–8 |
| Red blood cell transfusion | | | | | |
| | % | 22 | 60 | 74 | 60 |
| | Median units | 2 | 2 | 2 | 2 |
| | Range | 2–4 | 2–4 | 2–5 | 2–4 |
| Platelets transfusion | | | | | |
| | % | 4 | 25 | 37 | 47 |
| | Median units | 9 | 6 | 6 | 6 |
| | Range | 6–12 | 6–48 | 6–48 | 6–36 |
| Nausea – vomiting | | | | | |
| | Grade 2–3 (%) | 37 | 42 | 32 | 19 |
| | Grade 4 (%) | 0 | 0 | 2 | 2 |
| Mucositis | | | | | |
| | Grade 2–3 (%) | 8 | 17 | 19 | 14 |
| | Grade 4 (%) | 2 | 0 | 2 | 0 |
| Liver toxicity | | | | | |
| | Grade 1–2 (%) | 33 | 19 | 17 | 19 |
| | Grade 3 (%) | 0 | 0 | 0 | 0 |

**Table 4** Response rate in the 48 stage IV patients

| | | *n* | % |
|---|---|---|---|
| Complete response | | 7 | 14.5 |
| Partial response | | 24 | 50 |
| Overall response | | 31 | 64.5 |
| Stable disease | | 12 | 25 |
| Progressive disease | | 2 | |
| Objective response by site | Liver | 16 | 84 |
| | Lymph nodes | 16 | 84 |
| | Pleura | 2 | 67 |
| | Lung | 5 | 56 |
| | CNS | 2 | 67 |

CI: 51–78) with a 14.5% complete response rate of 14.5% (95% CI: 4.5–24.5). Response rate according to metastatic site is shown in Table 4. Only 2 patients (4%) experienced outright progression at the end of the study regimen. Out of 6 evaluable inflammatory breast cancer patients, 5 presented clinical objective response. Overall response rate for the 56 patients was 64.3% (95% CI: 51.8–76.8). Pathological response was assessed in breast and in axillary lymph nodes for the patients who underwent radical mastectomy and axillary lymph nodes dissection after induction chemotherapy. Breast pathological response was assessable in 19 patients (13 metastatic and 6 inflammatory). Complete response was observed in 2 patients (11%) and partial response in 4 patients (21%). 3 complete responses in axillary lymph node dissections (17%) were observed among 18 evaluable patients.

Median follow-up was 26 months for stage IV patients (range: 2–64). Survivals were computed from the first day of the first course. Intent-to-treat median progression-free survival for stage IV patients was 22.5 months. Median OS for stage IV patients was 37 months. Median OS for the 56 patients was 39 months (Figure 1). Median OS for stage IV patients with visceral metastases was 27 months (26 months in the 19 pts with liver metastases), and 48 months for patients with non-visceral metastases (*P* = 0.04). Median PFS was, respectively, 20 months and 42 months (*P* = 0.04). As of January 31st, 2000, 3 stage IIIB patients had progressed at 4, 5 and 28 months. The 5 other patients are disease-free at 27+, 30+, 39+, 41+ and 43+ months.

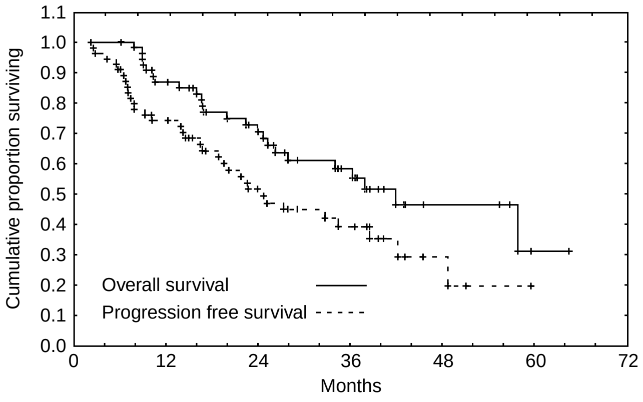

**Figure 1** Overall and progression-free survival in the 56 patients

*© 2001 Cancer Research Campaign*

### DISCUSSION

On the basis of so far 3 reported randomized studies (Peters et al, 1996; Lotz et al, 1999; Stadtmauer et al, 2000), the value and feasibility of HDC in metastatic breast cancer has been extensively and critically reviewed (Antman, 1999; Crown et al, 1999; Gradishar, 1999; Hortobagyi, 1999; Livingston and Crowley, 1999; MacNeil and Eisenhauer, 1999). The negative PBT-1 trial (Stadtmauer et al, 2000), which included the largest number of patients, has been widely commented: from 553 patients initially included in the study, only 199 (of whom only 45 CR patients) were amenable to randomization. The imbalance between the prognostic factors of the 2 arms, favouring the control arm, has also been questioned (Antman, 1999), as well as the concern about the *standard* arm of continuous CMF (MacNeil and Eisenhauer, 1999). The trial thus suffered a spectacular attrition, and lost much statistical power (Gradishar, 1999; Hortobagyi, 1999; Livingston and Crowley, 1999). The two other studies (Lotz et al, 1999; Peters et al, 1996) showed either significant differences or a trend for an advantage for HDC. One of them included mitoxantrone, an anthracyclin derivative, in the high-dose regimen, but with few patients (Lotz et al, 1999), and one proposed upfront high-dose chemotherapy in the experimental arm (Peters et al, 1996).

The conditioning regimen of these studies varied in types of drugs, doses and haematopoietic support. The optimal regimen and schedule of HDC remains thus a striking question deserving well designed trials, as underscored by all editorialists (Antman, 1999; Crown et al, 1999; Hortobagyi, 1999; Livingston and Crowley, 1999; MacNeil and Eisenhauer, 1999). Another issue is the determination of the characteristics of the patients likely to benefit from HDC, with selection of patients for HDC trials being another major problem. A review of 1581 metastatic patients treated at the M.D Anderson Cancer Center with doxorubicin-based regimens has shown that patients who presented conventional eligibility criteria for HDC trials (younger age, performance status ≤ 2, chemosensitivity) also had the best prognosis, with median OS and PFS of 30 and 16 months respectively (Rahman et al, 1997). These results had been shown after stratification according to previously described prognostic factors (Ayash et al, 1995) and appear to be in the same range as the best results of HDC trials. It is of note that extent of disease (number of metastatic site and visceral involvement) did not differ between HDC candidates and non-candidates, and was not included in the prognostic analyses. Adverse prognostic factors for patients entering HDC trials include previous adjuvant chemotherapy, liver metastases, extensive metastatic disease and chemoresistant disease (Ayash et al, 1995; Rizzieri et al, 1999; Rowlings et al, 1999).

The present study tried to circumvent some of the factors limiting the interpretation of the presently available HDC trials for metastatic breast cancer. We first established the feasibility of a multicycle, epirubicin-containing, high-dose regimen and the criteria we had set were all met. Leukapheresis was efficient, as more than $4 \times 10^6$ kg$^{-1}$ CD34+ PBSC were collected in 54 patients (96%, objective: 80%), and 1 or 2 leukapheresis sessions were sufficient in 53 patients (94%). The dose-intensity was greater than 80% in 45 out of the 48 patients (94%) who received the 4 courses (objective: 80%). 8 patients (16%) were withdrawn from the study because of toxicity, though no toxicity-related death was observed. 4 of these 8 patients had severe infectious complications, 3 had profound asthenia, and 1 haemorrhagic cystitis. No long-term side effect was recorded; there was no cardiac function deterioration, nor clinical cardiotoxicity detected. Most important, surgery, radiation therapy, maintenance and second-line chemotherapies were administered without unusual toxicity. The patients who were included in the study presented many adverse prognostic factors (Table 1), and were not preselected by the chemosensitivity of their disease. Objective response was conservatively assessed with a rate of 69% (intent to treat: 64.5%), and a complete response rate of 16% (intent to treat: 14.5%). These results have to be compared to those obtained with standard-dose chemotherapy (reviewed in Flamm Honig, 1996). More recent drugs have also been included in combination therapies, yielding very promising results that may compare favourably with high-dose chemotherapy (Dieras et al, 1996; Dieras, 1998). It is also of note that escalating the dose of cyclophosphamide has not been beneficial in the adjuvant setting (Fisher et al, 1997, 1999). However, we report here a median overall survival (37 months) and median progression-free survival (22.5 months) that are in the high end of reported data for patients undergoing high-dose chemotherapy as consolidation, with similar follow-up (Peters et al, 1996; Antman et al, 1997; Lotz et al, 1999; Rowlings et al, 1999; Stadtmauer et al, 2000). Moreover, only 53% and 25% of the ABMTR patients had visceral (32% liver) and extensive disease, respectively (Rowlings et al, 1999), compared to 65% and 67% in the present study, respectively. The choice of epirubicin over doxorubicin has contributed to the feasibility of our approach. Its higher clearance, resulting from glucuronidation (Coukell and Faulds, 1997), is probably at the root of its briefer haematotoxicity, and has allowed the dose-intense, repetitive regimen we report here.

The reappraisal of HDC in metastatic breast cancer patients, combined with the encouraging results of the present study which included young patients with very poor prognosis, gives important clues to the potential role of HDC in metastatic breast cancer patients, yet to be explored for an eventual therapeutic contribution. High-dose chemotherapy with haematopoietic support should be administered early, in a multicycle outpatient programme, and should be epirubicine based. Several questions remain open, such as the eventual role of taxanes in HDC protocols, even if a dose–response effect has not been clearly demonstrated for this family of cytotoxic agents (Hryniuk et al, 1998), and the role of newly available therapies such as anti Her2/neu antibodies. Large clinically relevant and well designed cooperative randomized trials addressing these different issues are still awaited.

### ACKNOWLEDGEMENTS

We would like to thank Dr C Hudis and Pr E Cvitkovic for their critical reading and comments, and Ms M Hughes for her editorial assistance.

### REFERENCES

Antman K (1999) Critique of the high dose chemotherapy studies in breast cancer: a positive look at the data. *J Clin Oncol* **17**: 30–35

Antman KH, Rowlings PA, Vaughan WP, Pelz CJ, Fay JW, Fields KK, Freytes CO, Gale RP, Hillner BE, Holland HK, Kennedy MJ, Klein JP, Lazarus HM, McCarthy PL, Jr, Saez R, Spitzer G, Stadtmauer EA, Williams SF, Wolff S, Sobocinski KA, Armitage JO and Horowitz MM (1997) High-dose chemotherapy with autologous hematopoietic stem-cell support for breast cancer in North America. *J Clin Oncol* **15**: 1870–1879

Ayash L, Wheeler C, Fairclough D, Schwartz G, Reich E, Warren D, Schnipper D, Antman K, Frei III E and Elias A (1995) Prognostic factors for prolonged

*© 2001 Cancer Research Campaign* 

progression free survival with high dose chemotherapy with autologous stem cell support for advanced breast cancer. *J Clin Oncol* **13**: 2043–2049

Bastholt L, Dalmark M, Gjedde S, Pfeiffer P, Pedersen D, Sandberg E, Kjaer M, Mouridsen H, Rose C, Nielsen O, Jakobsen P and Bentzen S (1996) Dose–response relationship of epirubicin in the treatment of postmenopausal patients with metastatic breast cancer: A randomized study of epirubicin at four different dose levels performed by the Danish Cancer Cooperative Group. *J Clin Oncol* **14**: 1146–1155

Bezwoda W, Seymour L and Dansey R (1995) High-Dose Chemotherapy With Hematopoietic Rescue as Primary Treatment for Metastatic Breast Cancer. *J Clin Oncol* **13**: 2483–2489

Cottu P, Zelek L, Extra J, Espié M, Mignot L, Morvan F and Marty M (1999) High-Dose epirubicin and cyclophosphamide every two weeks as first line chemotherapy for relapsing metastatic breast cancer patients. *Ann Oncol* **10**: 795–801

Coukell A and Faulds D (1997) Epirubicin. An Updated Review of its Pharmacodynamics and Pharmacokinetics Properties and Therapeutic Efficacy in the Management of Breast Cancer. *Drugs* **53**: 453–482

Crown J, Coiffier B, Cortès-Funes H, Guillaume T, Kanz L, Kvalheim G, Marty M and Symann M (1999) ESTIC position paper: high-dose chemotherapy for breast cancer, investigation should continue. *Ann Oncol* **10**: 903–905

Culine S, Extra JM, Espie M, Bourstyn E, Dieras V, Giacchetti S, Maylin C, De Rocquancourt A, Morvan F and Marty M (1993) [Promising results of bimonthly chemotherapy with high dose cyclophosphamide and epirubicin in induction treatment of inflammatory cancer of the breast]. *Bull Cancer (Paris)* **80**: 994–1000

Dal Cortivo L, Cottu P, Lotz J, Robert I, Extra J, Miclea J, Marty M and Marolleau J (2001) Residual tumor cell in peripheral blood stem cells collections of 117 breast cancer patients evaluated by immunocytochemical technique. *J Hematother Stem Cell Res* in press.

Dieras V (1998) Taxanes in combination with doxorubicin in the treatment of metastatic breast cancer. *Semin Oncol* **25**: 18–22

Dieras V, Extra J, Bellissant E, Espié M, Morvan F, Pierga J, Mignot L, Tresca P and Marty M (1996) Efficacy and Tolerance of Vinorelbine and Fluorouracile Combination as First-Line Chemotherapy of Advanced Breast Cancer: Results of a Phase II Study Using a Sequential Group Method. *J Clin Oncol* **14**: 3097–3104

Extra J, Rousseau F, Mignot L, Espié M, Morvan F and Marty M (1995) High-dose induction chemotherapy (HDIC) in breast cancer with metastases (MBC) at diagnosis. *Proc Ann Meet Am Soc Clin Oncol*

Fisher B, Anderson S, Wickerham DL, DeCillis A, Dimitrov N, Mamounas E, Wolmark N, Pugh R, Atkins JN, Meyers FJ, Abramson N, Wolter J, Bornstein RS, Levy L, Romond EH, Caggiano V, Grimaldi M, Jochimsen P and Deckers P (1997) Increased intensification and total dose of cyclophosphamide in a doxorubicin-cyclophosphamide regimen for the treatment of primary breast cancer: findings from National Surgical Adjuvant Breast and Bowel Project B-22. *J Clin Oncol* **15**: 1858–1869

Fisher B, Anderson S, DeCillis A, Dimitrov N, Atkins JN, Fehrenbacher L, Henry PH, Romond EH, Lanier KS, Davila E, Kardinal CG, Laufman L, Pierce HI, Abramson N, Keller AM, Hamm JT, Wickerham DL, Begovic M, Tan-Chiu E, Tian W and Wolmark N (1999) Further evaluation of intensified and increased total dose of cyclophosphamide for the treatment of primary breast cancer: findings from National Surgical Adjuvant Breast and Bowel Project B-25. *J Clin Oncol* **17**: 3374–3388

Flamm Honig S (1996) Hormonal therapy and chemotherapy. In *Diseases of the breast*, Harris J, Lippmann M, Morrow M and Hellman S (eds) pp 669–734. Lippincott-Raven: Philadelphia

Gilewski T and Norton L (1996) Cytokinetics and Breast Cancer Chemotherapy. In *Diseases of the Breast*, Harris J, Lippman M, Morrow M and Hellman S (eds) pp 751–767. Lippincott-Raven: Philadelphia

Gradishar W (1999) High dose chemotherapy and breast cancer. *JAMA* **282**: 1378–1380

Hortobagyi G (1999) Evolving concepts in high-dose chemotherapy for breast cancer. In *1999 Fall Educational Book* pp 128–134. American Society of Clinical Oncology: Alexandria.

Hryniuk W, Frei III E and Wright F (1998) A single scale for comparing dose-intensity of all chemotherapy regimens in breast cancer: summation dose-intensity. *J Clin Oncol* **16**: 3137–3147

Kaplan E and Meier P (1958) Non parametric estimation from incomplete observations. *J Am Stat Assoc* **53**: 457–481

Lalisang R, Wils J, Nortier H, Burghouts J, Hupperets P, Erdkamp F, Schouten H and Blijham G (1997) Comparative Study of Dose escalation Versus Interval Reduction to Obtain dose-Intensification of Epirubicin and Cyclophosphamide with Granulocyte Colony-Stimulating Factor in Advanced Breast Cancer. *J Clin Oncol* **15**: 1367–1376

Livingston R and Crowley J (1999) Commentary on PBT-1 study of high dose consolidation versus standard therapy in metastatic breast cancer. *J Clin Oncol* **17**: 22–24

Lotz J, Curé H, Janvier M, Morvan F, Asselain B, Guillemot M, Laadem A, Maraninchi D, Gisselbrecht C and Roché H (1999) High-dose chemotherapy with hematopoietic stem cells transplantation for metastatic breast cancer: results of the French Protocol PEGASE 04. In *Am Soc Clin Oncol* Vol. 18. pp 43a (abstract 161): Atlanta, GA

MacNeil M and Eisenhauer E (1999) High dose chemotherapy: is it standard management for any common solid tumor? *Ann Oncol* **10**: 1145–1161

Peters W, Jones R, Vredenburgh J, Shpall E, Hussein A, Elkordy M, Rubin P, Ross M and Berry D (1996) A large, prospective, randomized trial of high-dose combination alkylating agents (CPB) with autlogous cellular support (ABMS) as consolidation for patients with metastatic breast cancer achieving complete remission after intensive doxorubicin-based induction therapy. In *Am Soc Clin Oncol* Vol 15. pp 121

Piccart M, Bruning P, Wildiers J, Awada A, Schornagel J, Thomas J, Tomiak E, Bartholomeus S, Witteveen P and Paridaens R (1995) An EORTC pilot study of filgrastim (recombinant human granulocyte colony stimulating factor) as support to a high dose-intensive epiadriamycin-cyclophosphamide regimen in chemotherapy-naive patients with locally advanced or metastatic breast cancer. *Ann Oncol* **6**: 673–677

Rahman ZU, Frye DK, Buzdar AU, Smith TL, Asmar L, Champlin RE and Hortobagyi GN (1997) Impact of selection process on response rate and long-term survival of potential high-dose chemotherapy candidates treated with standard-dose doxorubicin-containing chemotherapy in patients with metastatic breast cancer [see comments]. *J Clin Oncol* **15**: 3171–3177

Rizzieri D, Vredenburgh J, Jones R, Ross M, Shpall E, Hussein A, Broadwater G, Berry D, Petros W, Gilbert C, Affronti M, Coniglio D, Rubin P, Elkordy M, Long G, Chao N and Peters W (1999) Prognostic and predictive factors for patients with metastatic breast cancer undergoing aggressive induction therapy followed by high dose chemotherapy with autologous stem cell support. *J Clin Oncol* **17**: 3064–3074

Rodenhuis S, Westermann A, Holtkamp M, Nooijen W, Baars J, van der Wall E, Slaper-Cortenbach I and Schornagel J (1996) Feasibility of multiple courses of high dose cyclophosphamide, thiotepa, and carboplatin for breast cancer or germ cell cancer. *J Clin Oncol* **14**: 1473–1483

Rowlings P, Williams S, Antman K, Fields K, Fay J, Reed E, Pelz C, Klein J, Sobocinski K, Kennedy M, Freytes C, McCarthy Jr P, Herzig R, Stadtmauer E, Lazarus H, Pecora A, Bitran J, Wolff S, Gale R, Armitage J, Vaughan W, Spitzer G and Horowitz M (1999) Factors correlated with progresion free survival after high dose chemotherapy and hematopoietic stem cell transplantation for metastatic breast cancer. *JAMA* **282**: 1335–1343

Shapiro C, Ayash L, Webb I, Gelman R, Keating J, Williams L, Demetri G, Clark P, Elias A, Duggan D, Hayes D, Hurd D and Henderson I (1997) Repetitive Cycles of Cyclophosphamide, Thiotepa, and Carboplatin Intensification With Peripheral-Blood Progenitor Cells and Filgrastim in Advanced Breast Cancer Patients. *J Clin Oncol* **15**: 674–683

Skipper H, Schabel F and Wilcox W (1964) Experimental evaluation of potential anticancer agents. XIII. On the criteria and kinetics associated with curability of leukemia. *Cancer Chemotherapy Research* **35**: 1–12

Snedecor G and Cochran W (1989) *Statistical methods*. Iowa State University Press: Ames, Iowa

Stadtmauer E, O'Neill A, Goldstein L, Crilley P, Mangan K, Ingle J, Brodsky I, Martino S, Lazarus H, Erban J, Sickles C and Glick J (2000) Conventional dose chemotherapy compared with high-dose chemotherapy plus autologous hematopoietic stem-cell transplantation for metastatic breast cancer. *N Eng J Med* **342**: 1069–1076

Vahdat L and Antman K (1997) High-Dose chemotherapy with autologous stem cell support for breast cancer. *Curr Opin Hematol* **4**: 381–389

Weiss RB, Gill GG and Hudis CA (2001) An on-site audit of the south african trial of high-dose chemotherapy for metastatic breast cancer and associated publications. *J Clin Oncol* **19**: 2771–2777

*© 2001 Cancer Research Campaign*
